# A Simple Algorithm for Immediate Postmastectomy Reconstruction of the Small Breast—A Single Surgeon's 10-Year Experience

**Published:** 2012-12-10

**Authors:** Magelia Kitcat, Alexandra Molina, Charlotte Meldon, Nagham Darhouse, Jon Clibbon, Charles M. Malata

**Affiliations:** ^a^Department of Plastic and Reconstructive Surgery, Addenbrooke's Hospital, Cambridge University Hospitals NHS Foundation Trust, Cambridge, United Kingdom; ^b^School of Clinical Medicine, University of Cambridge, Cambridge, United Kingdom; ^c^Norfolk & Norwich University Hospital, Norwich, United Kingdom; ^d^Cambridge Breast Unit, Addenbrooke's Hospital, Cambridge University Hospitals NHS Foundation Trust, Cambridge, United Kingdom

## Abstract

**Introduction:** Immediate small breast reconstruction poses challenges including limited potential donor site tissues, a thinner skin envelope, and limited implant choice. Few patients are suitable for autologous reconstruction while contralateral symmetrization surgery that often offsets the problem of obvious asymmetry in thin and small-breasted patients is often unavailable, too expensive, or declined by the patient. **Methods:** We reviewed 42 consecutive patients with mastectomy weights of 350 g or less (the lowest quartile of all reconstructions). Indications for the mastectomy, body mass index, bra cup size, comorbidity, reconstruction type, and complications were recorded. **Results:** A total of 59 immediate reconstructions, including 25 latissimus dorsi flaps, 23 implant-only reconstructions, 9 abdominal flaps, and 2 gluteal flaps, were performed in 42 patients. Of the 42 mastectomies, 4 were prophylactic. Forty-three percent of patients had immediate contralateral balancing surgery. The average mastectomy weight was 231 g (range, 74-350 g). Seven percent of implant-based reconstructions developed capsular contracture requiring further surgery. One free transverse rectus abdominus myocutaneous flap failed because of fulminant methicillin resistant staphylococcus aureus septicaemia. **Discussion and Conclusion:** Balancing contralateral surgery is key in achieving excellent symmetry in reconstruction small-breasted patients. However, many patients wish to avoid contralateral surgery, thus restricting a surgeon's reconstructive options. Autologous flaps, traditionally, had not been considered in thinner women because of inadequacy of donor site tissue, but in fact, often, as with larger-breasted patients, produce superior cosmetic results. We propose a simple algorithm for the reconstruction of small-breasted women (without resorting to super-complex microsurgery), which is designed to tailor the choice of reconstructive technique to the requirements of the individual patient.

Breast reconstruction in thin and small-breasted women presents specific challenges to the reconstructive plastic surgeon. Foremost among these is the low body mass index (BMI), which limits the number of autologous tissue donor sites available. It would, therefore, seem appropriate to choose prostheses as the first option for reconstruction. However, the use of expanders and implants in thin patients can also be unsatisfactory, often resulting in palpable or visible rippling due to the thinner skin and soft tissue envelope. The breast may also be so small that prosthetic or autologous reconstruction may result in a mound that is larger than the contralateral breast. Furthermore, any size mismatch is likely to be more noticeable in patients with small breasts. This situation can be rectified by contralateral simultaneous or sequential balancing breast augmentation^1^ Similarly, contralateral mastopexy may be used to improve symmetry. Understandably, however, some patients are unwilling to undergo an operation on their one remaining normal breast. The use of a latissimus dorsi (LD) myocutaneous flap with an implant is conventionally the standard choice in this circumstance.^2^ Other alternative autologous reconstructive options include abdominal free flaps^3^ or the newer gluteal artery perforator (GAP)^4^ and transverse upper gracilis flaps.^5^^,^^6^ These options, however, are donor site dependent.

The patient's expectations of breast reconstruction should be discussed fully including the possible aesthetic outcomes in light of their specific body morphology. The aim is to have a well-informed patient who goes on to achieve the optimum aesthetic outcome with minimal morbidity. After a review of our experience with different reconstructive techniques in small-breasted patients, we proposed a simple algorithm that could easily be applied to thin small-breasted patients (Fig 1).

## PATIENTS AND METHODS

### Definitions

For the purposes of this study, a small breast was arbitrarily defined as one with a mastectomy weight of less than 350 g. This comprised the lower quartile of all mastectomy weights. Clinically this usually translated to a UK bra cup size of B or smaller.

### Patient population and methods

All patients undergoing immediate breast reconstruction between 2000 and 2009 under the care of a consultant plastic surgeon with a specialist interest in breast reconstruction (C.M.M.) were identified from the theatre records, consultant's log book, and histopathology reports. Surgery was performed at a tertiary university teaching hospital. Patients meeting the criterion mentioned earlier were identified from the intraoperative mastectomy weights and the histopathology reports. All charts were available for review.

The features of interest to the study were the mastectomy type, reconstructive method, timing with respect to the mastectomy, any simultaneous contralateral balancing surgery undertaken, subsequent symmetrization surgery, any revisional surgery required, and the reconstructive outcomes. All patients were followed up for a minimum of 1 year. This allowed the authors to review the oncological outcomes such as disease recurrence and survival as well as the settled aesthetic outcome including the effects of radiotherapy and the result of revisional and delayed contralateral balancing surgery, if any.

## RESULTS

Over the 10-year period, 266 patients underwent immediate breast reconstruction, of whom 42 (16%) had mastectomy weight(s) less than 350 g and constituted the study group. Their median mastectomy weight was 225 g (range, 74-348 g). The small-breasted patients were aged 39 to 64 years with a median age of 50 years, and their mean BMI was 22. The UK bra cup sizes were A, 43%; B, 24%, and unspecified, 33%.

The indications for the mastectomies were risk reduction (4), ductal carcinoma in situ (16), and invasive carcinomas (22). The latter comprised ductal (14), lobular (3), no specific type (4), and mucinous (1). All invasive cancer patients went on to receive adjuvant chemotherapy. Their oncological outcomes are summarized in Table 1.

A total of 59 reconstructions were performed in the 42 patients with small breasts (Table 2). The reconstructions undertaken were 23 implant-only, 25 LD flaps (of which 4 were totally autologous), 8 free, and 1 pedicled abdominal flaps, and 1 superior gluteal artery perforator (SGAP) flap and 1 inferior gluteal artery perforator (IGAP) flap.

Eighteen of the 42 patients (43%) underwent bilateral immediate reconstructions. Of the 24 unilateral reconstructions, 3 (12%) had delayed contralateral balancing surgery; thus, overall 50% of the study patients had surgery to both breasts. Contralateral immediate symmetrization surgery included implant-only reconstruction (11), LD with implant reconstructions (3), and mastopexy (4). Delayed contralateral balancing surgery included implant only (2) and LD only (1).

One free transverse rectus abdominus myocutaneous (TRAM) flap failed because of fulminant methicillin resistant staphylococcus aureus septicemia on postoperative day 5 (an hour before she was due to be discharged from hospital), and salvage reconstruction was successfully performed 8 months later using an LD flap and expandable implant. Four patients had severe capsular contractures, which necessitated capsulectomy and implant exchange at a later date. One infected implant was removed, but the patient declined further reconstructive surgery. The majority of the patients achieved satisfactory aesthetic results. A small proportion of patients have required revisional surgery (Table 3).

## DISCUSSION

**Figure 1:**
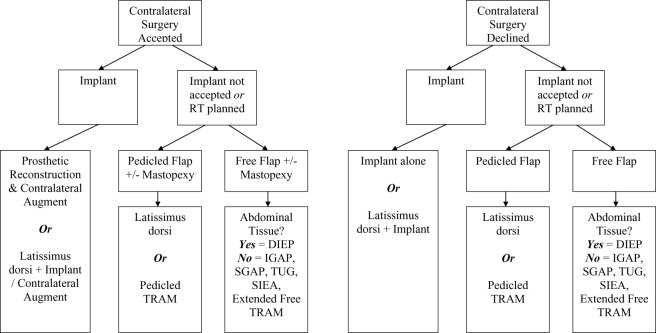
The reconstructive algorithm for small breast immediate reconstruction. RT indicates radiotherapy; DIEP, deep inferior epigastric artery perforator; IGAP, inferior gluteal artery perforator; SGAP, superior gluteal artery perforator; TUG, transverse upper gracilis; SIEA, superficial inferior epigastric artery; TRAM, transverse rectus abdominus myocutaneous.

Small breast reconstruction can be challenging but need not be complicated. Often patients are delighted that, despite their small breast size, there are still a number of available reconstructive options. We have designed a simple algorithm (Fig 1) to assist the reconstructive breast surgeon in selecting a suitable option for a patient in order to achieve a cosmetically acceptable result in terms of both symmetry and shape.

In our experience, when contralateral balancing surgery is accepted by the patient, implant reconstruction is the simplest option. We were able to achieve good symmetry with either expandable implants or fixed volume implants in both breasts. The senior author's preferred technique is subpectoral implant insertion. The use of a pedicled LD flap, in addition to the prosthesis, offsets the problem of rippling to some degree, as the muscle can be draped to cover a large part, if not all, of the implant surface.

However, if the patient declines the use of implants or if radiotherapy is planned postoperatively, pedicled flaps such as LD or TRAM with or without contralateral mastopexy can give outstanding results. Another alternative is the use of a free flap. This is, however, donor site dependent especially in this group of patients. If there is adequate abdominal tissue, a free deep inferior epigastric artery perforator flap can give an excellent result. A limited number of patients, in our study, were suitable for this reconstruction type. If there was insufficient abdominal tissue, we opted to use an IGAP or SGAP flap. Some authors also advocate the use of a transverse upper gracilis or transverse myocutaneous gracilis flap,^5^^,^^6^ superficial inferior epigastric artery, or extended free TRAM. Achieving excellent results, when contralateral surgery is declined, is still possible using the algorithm, but careful selection of the patient is even more important.

Traditionally, autologous tissue reconstruction has been thought to be challenging in thin women because of the lack of adequate donor tissue. Prosthesis-only reconstruction is, therefore, commonly used for reconstructing small breasts and may entail the use of simple implants, traditional expanders, or expandable implants.^7^^,^^8^ Previous or planned radiotherapy is, of course, a relative contraindication to prosthetic reconstruction. Implant shapes can be either anatomical or round, with round implants tending to create a breast mound with low projection, excessive upper pole fullness, and upper pole rippling, which is particularly noticeable in thin patients. This makes anatomical implants preferable to round ones, generally, in breast reconstruction. However, round implants may be useful to match a small contralateral breast with minimal projection or in a patient with flat round breasts. Following skin-sparing mastectomy, a simple circumareolar purse-string reconstruction using implants has been described for patients with small nonptotic breasts and allows the conical shape of the breast to be preserved in a single-stage procedure.^9^

**Figure 2:**
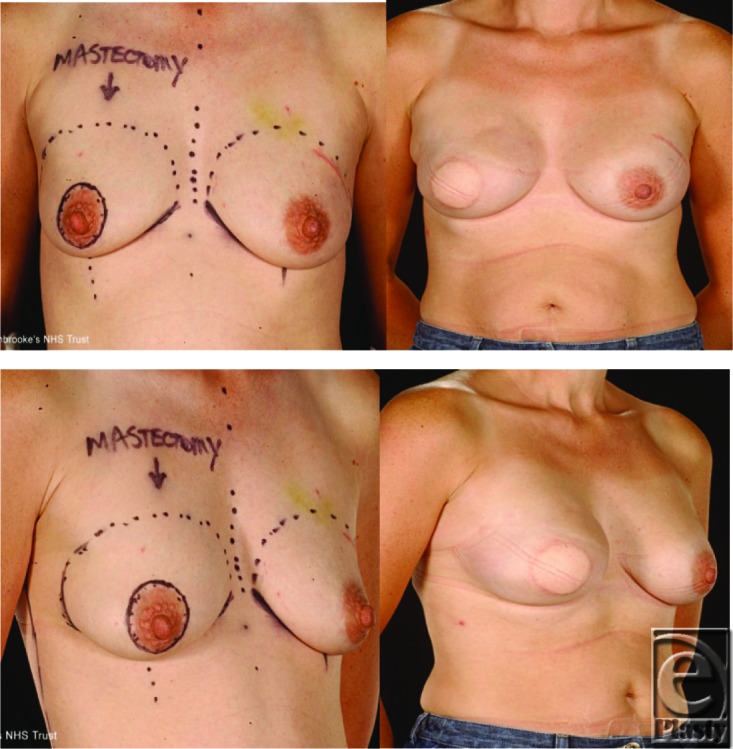
A 41-year-old woman with B cup breasts had immediate reconstruction with a latissimus dorsi flap and expandable implant with simultaneous contralateral augmentation. She declined nipple reconstruction.

**Figure 3:**
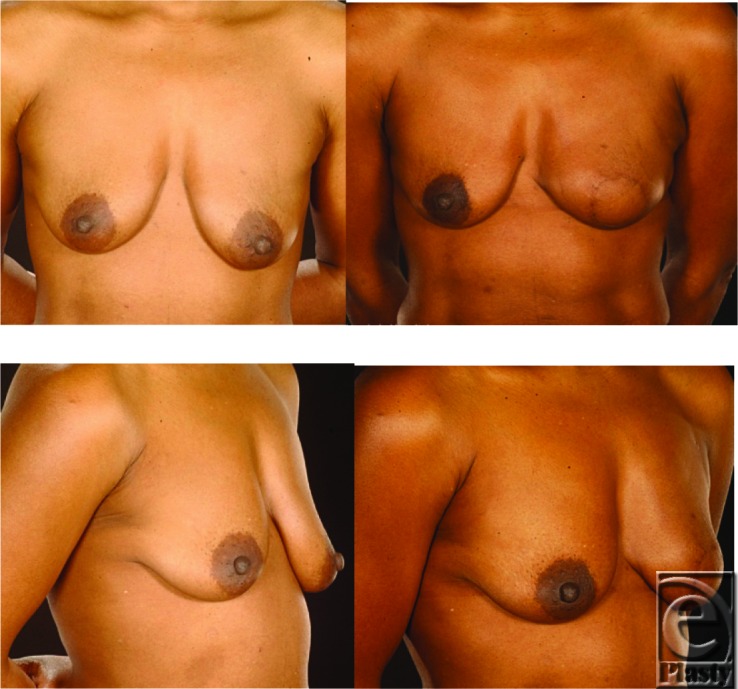
This 58-year-old woman with grade 2 ptotic breasts was reconstructed with a totally autologous latissimus dorsi flap. She declined contralateral surgery and refused to have implant augmentation. Please note the excellent shape and symmetry.

The LD flap is implant-assisted in most patients but can also be used successfully as a totally autologous breast reconstruction.^10^ The same author also described that the totally autologous LD or the so-called extended LD myocutaneous flap can be suitable in heavier-weight patients with BMI of 31.8. However, the extended LD can also be useful to reconstruct the small breasts of thin patients such as those in our series with an average BMI of 22.1. An alternative way of using the LD flap in thin patients is to raise the muscle only without a skin paddle. The muscle provides additional coverage of the prosthesis and the lack of the skin paddle avoids an external donor site scar.^11^ If using prostheses with LD flaps in thin women, we recommend that these should be placed deep to the pectoral muscle as well as beneath the flap to avoid palpable implant edges (Fig 2). We used the totally autologous LD flap in 4 patients with excellent results and minimal donor site morbidity (Fig 3).

**Figure 4:**
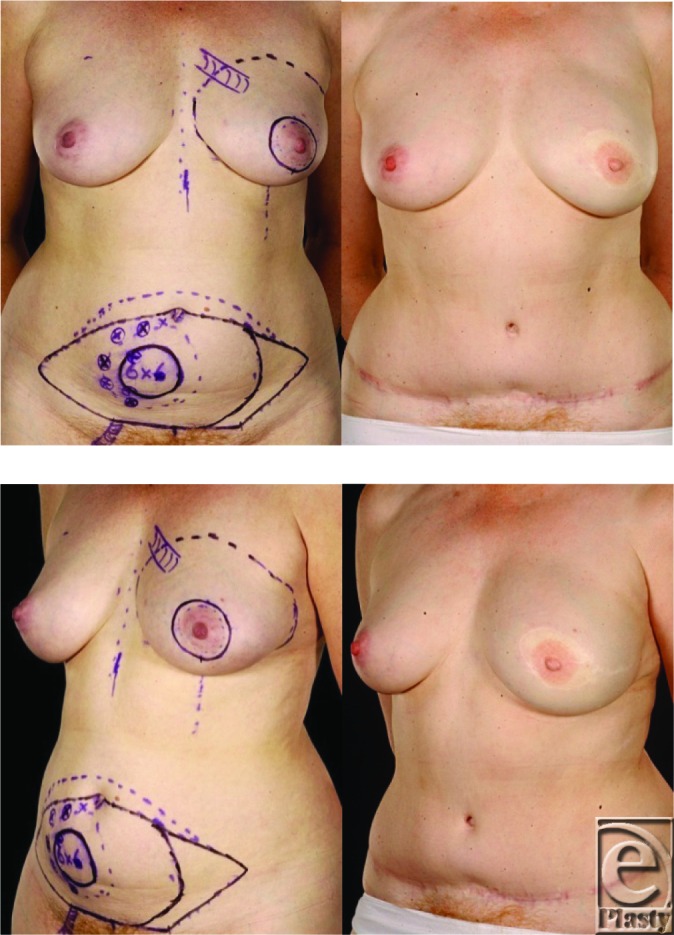
This 50-year-old woman had invasive mucinous cancer of her left breast. She had reconstruction with a free muscle-sparing TRAM flap using a single pedicled anastomosis; no contralateral surgery was needed. She had no recurrence on follow-up.

Despite assumptions that thin women generally have limited abdominal tissue, in carefully selected slim patients, superior cosmetic results can also be achieved with an abdominal free flap using a single pedicle anastomosis (Fig 4). Both pedicled and free TRAM flaps have been used successfully in slim women, who appear to have a better flap blood supply and thus a lower risk of complications.^12^ This allows the flap to be extended further laterally without increasing the incidence of partial flap loss or fat necrosis.^13^ By extending the flap as far laterally as possible and folding the flap over on itself, it is possible to create breast mounds with adequate projection even in thin patients. Kroll^13^ successfully used this technique to achieve bilateral reconstructions in 6 patients, all of whom were unusually thin. Even with such modifications, certain thin women do not, however, have sufficient abdominal tissue for a TRAM flap breast reconstruction. Therefore, it has been suggested that the TRAM flap should be combined with an implant in those women with inadequate subcutaneous abdominal fat.^14^^,^^15^ Women who benefit from combined TRAM flap and implant breast reconstruction include those with thin body habitus but relatively large ptotic breasts, and very thin women requesting bilateral reconstruction. Kronowitz et al^15^ studied 88 patients and found that TRAM and implant reconstructions were aesthetically more pleasing than LD and implants. We prefer to undertake implant augmentation of deep inferior epigastric artery perforator flaps as a delayed procedure^16^ contrary to the recommendations of others.^17^

**Figure 5:**
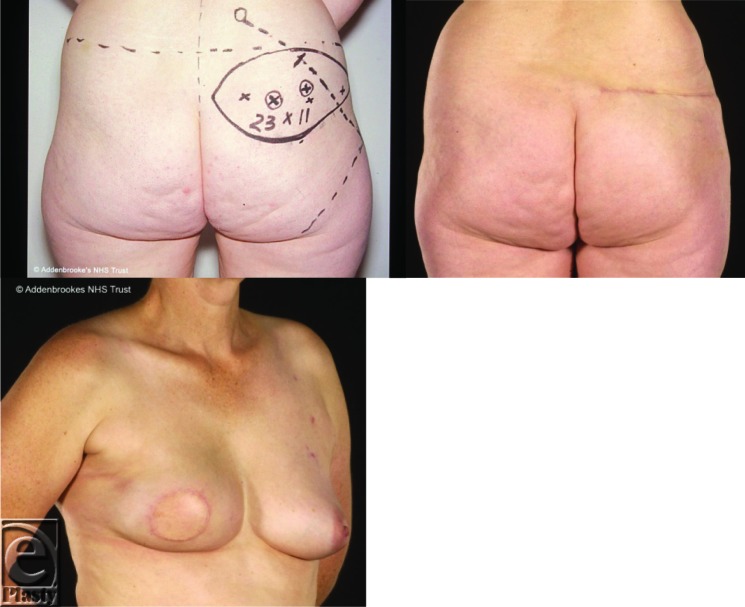
A superior gluteal artery perforator flap in a 48-year-old woman with a “flat” abdomen. She did not require contralateral surgery. Note that the main limitation of this flap is its short height.

**Figure 6:**
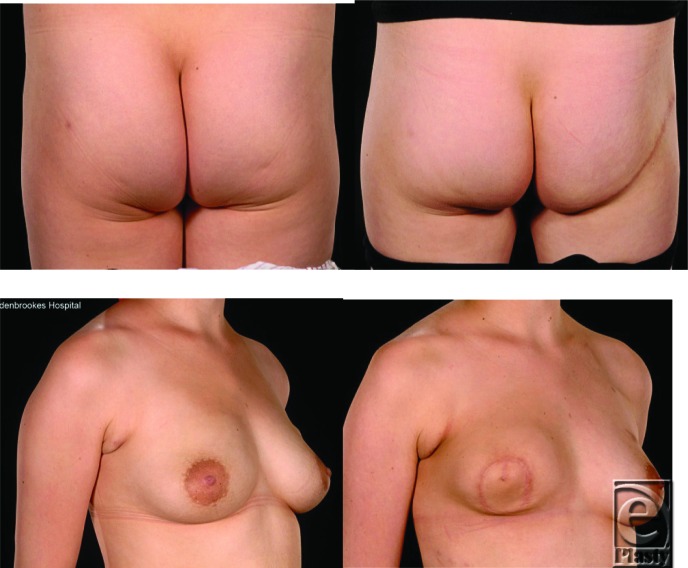
This 28-year-old woman had an inferior gluteal artery perforator (IGAP) flap reconstruction with no contralateral surgery. The patient had postoperative radiotherapy and note the radiation-induced flap contraction. She later underwent contralateral prophylactic mastectomy and immediate IGAP reconstruction (after this study period).

There are a number of less commonly performed flaps that may be useful in the small-breasted patient. The advantage of the SGAP flap specific to thin small-breasted women arises from the observation that there is a good match between the amount of tissue available in the superior gluteal region and the size of the breast to be reconstructed, even in thin women.^18^ In our study, our SGAP flaps has not only resulted in good shape and symmetry but had cosmetically acceptable donor site although we were able to use this flap in only 1 patient (Fig 5). This is because the usefulness of this flap is limited by the usually poor donor site and flap size restriction. The in-the-crease IGAP flap^19^ may prove to be more applicable and certainly has a better donor site (Fig 6). More recently, the transverse upper gracilis flap has been successfully used in reconstructing small- and moderate-sized breasts in patients who have sufficient thigh tissue.^20^ Its main disadvantage is the small flap volume, which means that an implant is needed more often than not.^6^

It is evident that slim, small-breasted women present unique challenges in breast reconstruction. The surgical approach must be tailored to the individual patient, and there are a number of newer autologous flap techniques that should be considered. Even in our small case series, a number of simple techniques were used successfully without resorting to complicated and highly technically challenging microsurgery. We would advocate involving the patient fully in all decisions regarding their breast reconstruction and using the algorithmic approach herein presented. The LD and implant technique remains our workhorse reconstruction in thin small-breasted patients with a thin body habitus.

## Figures and Tables

**Table 1:** Histology and outcomes of breast cancer patients

Histology	Number of patients	Outcome
Prophylactic	4	Disease free
DCIS (intermediate and high grade)	16	1 unrelated death
Invasive ductal carcinoma	14	2 died of disease
Invasive NST	4	2 lymph node spread
Invasive lobular carcinoma	3	1 died of disease
Invasive mucinous	1	Alive and disease-free

DCIS indicates ductal carcinoma in situ; NST, no specific type.

**Table 2:** Types of breast reconstruction

Type of reconstruction	Number of patients (*n* = 59)
Implant only	23
LD flap + implant	19
LD flap only (totally autologous)	6
Abdominal flaps	9
GAP flaps	2

LD indicates latissimus dorsi; GAP, gluteal artery perforator.

**Table 3:** Types and indications of revisional surgery

Subsequent Surgery	Type of Surgery/Indication
Balancing surgery	1 LD flap
	1 implant only
	1 implant and mastopexy
Revision of new breast	4 severe capsular contracture
	1 infection
Severe rippling	0

LD indicates latissimus dorsi.
